# Clinical heterogeneity of hyperornithinemia-hyperammonemia-homocitrullinuria syndrome in thirteen palestinian patients and report of a novel variant in the *SLC25A15* gene

**DOI:** 10.3389/fgene.2022.1004598

**Published:** 2022-11-24

**Authors:** Imad Dweikat, Reham Khalaf-Nazzal

**Affiliations:** ^1^ Metabolic Department, Faculty of Medicine, Arab American University of Palestine, Jenin, Palestine; ^2^ Department of Physiology and Medical Genetics, Faculty of Medicine, Arab American University of Palestine, Jenin, Palestine

**Keywords:** hyperornithinemia, hyperammonemia, homocitrullinuria, SLC25A15 gene, spasticity, hepatic failure, autosomal recessive, frameshift variant

## Abstract

**Background:** Hyperornithinemia-Hyperammonemia-Homocitrullinuria (HHH) syndrome, is a rare autosomal recessive disorder characterized by impaired ornithine transport across the inner mitochondrial membrane. HHH is caused by biallelic disease-causing variants in the *SLC25A15* gene. The clinical presentation of HHH is highly variable ranging from severe neonatal encephalopathy and hepatic failure to a milder form with corresponding learning difficulties.

**Methods:** In this study, data from thirteen patients with HHH syndrome, diagnosed between the age of 1 week–29 years at two tertiary care centers in Palestine, is presented. The clinical, biochemical, and molecular data are reviewed.

**Results:** Analysis of the *SLC25A15* gene sequence revealed a novel homozygous frameshift deletion in exon 5, NM_014252.4:c.552-555delTTTC; p (Phe185SerfsTer8) in nine patients. The remaining four patients had a recurrent homozygous frameshift variant; NM_014252.4:c.446delG, (p.Ser149ThrfsTer45). The major acute clinical presentation found was encephalopathy and liver dysfunction. Nervous system involvement was common, progressive, and presented with signs of upper motor neuron disease as well as variable degrees of cognitive impairment. One patient had an initial presentation in adulthood with acute encephalopathy that responded well to treatment. There was no clear genotype-phenotype correlation.

**Conclusion:** Our results confirm the marked clinical heterogeneity of HHH including severe neonatal presentation, hepatic failure, and progressive pyramidal tract dysfunction in all age groups. The disease progression was variable, even in patients with the same genetic variant, and in patients with severe neonatal-onset hepatic encephalopathy. We report a novel pathogenic variant in the *SLC25A15* gene, further expanding the molecular spectrum of the disease.

## Introduction

Hyperornithinemia-Hyperammonemia-Homocitrullinuria (HHH) syndrome (Phenotype MIM number 238970) is a rare genetic disease of the urea cycle caused by disease-causing variants in the Solute Carrier Family 25, Member 15 gene; *SLC25A15* (alias Ornithine Transporter, Mitochondrial, one; ORNT1, gene MIM number 603861) (Camacho et al., 1999; Tsujino et al., 2000; Salvi et al., 2001; Martinelli et al., 2015). Disease-causing variants in this gene result in impaired ornithine transport across the mitochondrial membrane; this interrupts the urea cycle and causes hyperammonemia. Impaired transport of ornithine causes its accumulation in the cytosol leading to hyperornithinemia (Oyanagi et al., 1983; Camacho et al., 1999). Inside the mitochondria, ornithine deficiency leads to the accumulation of carbamoylphosphate (Hommes et al., 1986; Inoue et al., 1988). The increased mitochondrial level of carbamoylphosphate results in either an excess production of orotic acid through the cytosolic pyrimidine biosynthetic pathway or the formation of homocitrulline from lysine by ornithine transcarbamylase (Panza et al., 2019). The combination of Hyperornithinemia, Hyperammonemia, and Homocitrullinuria is pathognomonic for HHH syndrome and is usually accompanied by the detection of increased levels of orotic acid in plasma or urine. However, initially, some patients may present with incomplete biochemical profiles such as normal plasma ornithine levels or minimal excretion of homocitrulline in urine (Shimizu et al., 1990; Martinelli et al., 2015).

HHH is characterized by marked phenotypic variability including age at onset, clinical presentation, and severity of symptoms. The most severe forms are those with neonatal onset of hypotonia, lethargy, and seizure progressing to coma and even death (Martinelli et al., 2015; Panza et al., 2019; Ramsey et al., 2022). HHH can present acutely in infancy, childhood, and adulthood with hyperammonemia, and liver dysfunction, or with a slowly progressive course including an aversion to protein-rich foods, mental regression, and signs of motor dysfunction (Hommes et al., 1986; Smith et al., 1992; Debray et al., 2008; Martinelli et al., 2015; Wild et al., 2019; Silfverberg et al., 2018; Panza et al., 2019; Ebrahimi-Fakhari et al., 2021). Even in patients homozygous for the same pathogenic variant, the clinical presentations and the outcome are variable (Debray et al., 2008).

Regardless of the age of onset and the type of the presentation, most patients develop signs of pyramidal tract dysfunction which vary from lower limb hyperreflexia, positive Babinski sign, and spastic gait to a full presentation of spastic paraparesis (Tsujino et al., 2000; Salvi et al., 2001; Debray et al., 2008; Martinelli et al., 2015). Moreover, there is no clear association between the neurological outcome and the age at diagnosis, delayed initiation of treatment, or relapses of hyperammonemia (Salvi et al., 2001; Debray et al., 2008). There is also often cerebellar dysfunction with ataxia, dysarthria, nystagmus, poor fine motor coordination, and tremor (Kim et al., 2012; Olivieri et al., 2019).

Herein, we describe the genotype, the phenotype, and the clinical course of thirteen Palestinian patients with HHH, followed at two tertiary care centers in Palestine over the past 10 years. The diagnosis was initially suggested biochemically by the combination of hyperammonemia and hyperornithinemia in most patients, or by the combination of hyperornithinemia and orotic aciduria in rare cases when ammonia level was within normal ranges. Homocitrullinuria was detected in six patients. Molecular genetic analysis of the *SLC25A15* gene confirmed the diagnosis in all patients.

## Subjects and methods

Informed consent for detailed clinical phenotyping, genetic studies, and publication of the results was obtained from the enrolled individuals and/or legal guardians (IRB approval was given by the Palestinian Health Research Council PHRC/HC/518/19) in compliance with the Declaration of Helsinki and local ethical committee guidance.

This is a retrospective analysis of thirteen Palestinian patients diagnosed with HHH syndrome based on clinical manifestation, hyperammonemia, quantitative plasma and urine amino acid analysis, qualitative urine organic acid analysis, and molecular genetic analysis. A detailed history was obtained from all patients or their families. Clinical examination was performed in each case at the time of the diagnosis and for twelve patients during follow-up at the metabolic clinic. One patient died at the age of 1 month due to severe encephalopathy and hepatic failure. Biochemical evaluations including full blood count, coagulation profile, liver transaminases, and abdominal ultrasound were performed on all patients at the time of the diagnosis and during follow-up every 4–6 months.

Blood samples for genetic testing were collected from all patients and their parents for DNA extraction using standard procedures. Direct sequencing of the *SLC25A15* exons and flanking intronic regions was performed using BigDye Terminator cycle sequencing, as previously described (Tsujino et al., 2000; Salvi et al., 2001).

## Results

### Genetic findings

In all recruited individuals, a molecular diagnosis of HHH syndrome was established along with the suggestive metabolic and biochemical findings. Dideoxy sequencing of the entire coding region and the flanking intronic boundaries identified two biallelic loss-of-function variants in the *SLC25A15* gene. All parents were heterozygous carriers for the detected variant in their children.

**FIGURE 1:**
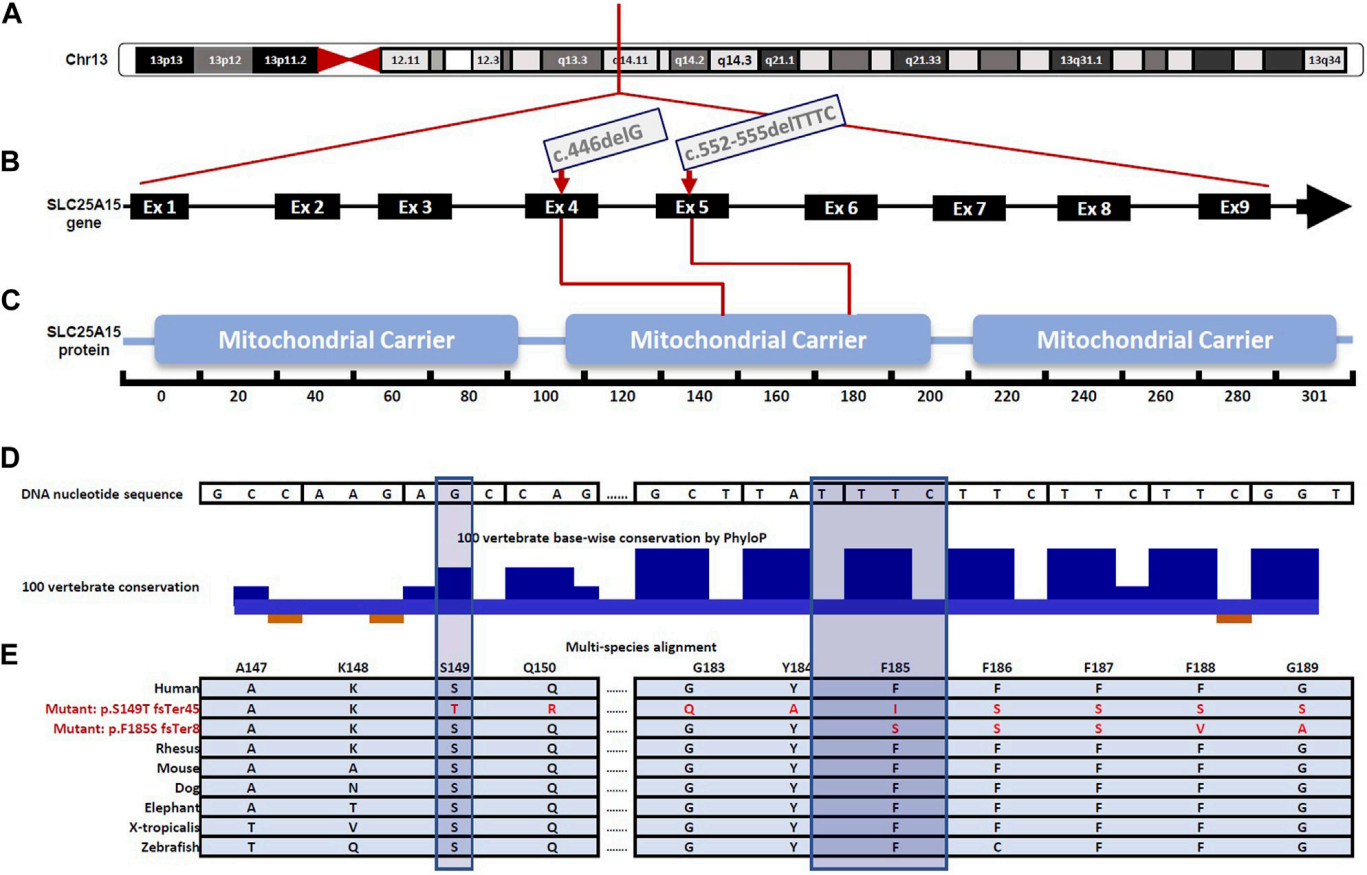
Schematic illustration for gene and protein domain structure of SLC25A15 highlighting variants identified in the Palestinian patient cohort affected by Hyperornithinemia-Hyperammonemia-Homocitrullinuria (HHH) Syndrome, **(A)** A simplified illustration of *SLC25A15* gene location on chromosome 13, specifically in the 13q14.11 region **(B)** Gene diagram showing the exon-intron organization of the *SLC25A15* gene (NNM_014252.4), comprised of 9 exons and is 2.93 kb in length. The c.446delG, (p.Ser149ThrfsTer45) and the c.552-555delTTTC, p (Phe185SerfsTer8) variants are located in exon 4 and exon 5, respectively (red arrows) **(C)** *SLC25A15* gene encodes a 301 amino acid protein predicted to contain three mitochondrial carrier domains, essential for the efficient transport of ornithine across the inner mitochondrial membrane during urea cycle **(D)** Zoomed view of the molecular region encompassing the p. Phe185SerfsTer8 and the p. Ser149ThrfsTer45 variants. The sites of the two variants are highly conserved among humans and other vertebrates as depicted using the PhyloP algorithm in the UCSC conservation track across 100 vertebrate species (shadowed bars indicate the amino acids where reading frame changes have occurred) **(E)** Multi-species alignment showing conservation of part of the molecular region encompassing the p (Phe185SerfsTer8) and p (Ser149ThrfsTer45) variants.

In nine patients (patients 1–9, Table 1), a novel homozygous frameshift variant in the *SLC25A15* gene, Chr13:g.41381529_41381532del, NM_014252.4:c.552-555delTTTC, p. (Phe185SerfsTer8) [GRCh37]; (Figures 1A–C); was identified. The variant is predicted to be deleterious in Mutation Taster (Schwarz et al., 2010), and ENTPRISE-X with a pathogenicity prediction score of 0.92 (Cutoff 0.5) (Zhou et al., 2018). The variant is rare and is absent in gnomAD (v2.1.1 and v3.1.1 datasets, July 2022). The variant is located in exon 5 (Figure 1B), and resulted in a phenylalanine to serine substitution at amino acid 185 (Figure 1E), and a frameshift and alteration of the following eight amino acids, followed by premature termination of translation (Figure 1E). The substituted phenylalanine corresponds to the first of four highly conserved consecutive TTC phenylalanine codons (nucleotides 553–564 in the cDNA) located at the end of the predicted fourth transmembrane helical domain of the SLC25A15 protein (Figures 1D,E). The frameshift-deletion variant is predicted to result in nonsense-mediated mRNA decay of the mutant transcript. This frameshift deletion is an equivalent deletion to a previously reported pathogenic variant in ClinVar (NM_014252.4:c.554_557del, ClinVar accession number VCV001325080), and is located within the same region of the four consecutive phenylalanine codons, where another founder pathogenic variant among French-Canadian HHH patients is reported (Camacho et al., 1999).

**TABLE 1:** Clinical phenotype, biochemical findings, and genotype of patients with HHH syndrome.

Patient number	Gender	Age at symptom onset	Age at diagnosis	Age at the recent evaluation	Consanguinity	Clinical manifestations, age of onset and clinical outcome	Serum ammonia	Plasma ornithine	Urine orotic acid (qualitative)	PT and INR^d^	AST^d^ IU/L (N < 60)	ALT^d^ IU/L (N < 60)	The variant
							µmol/L (normal <80)	µmol/L normal (10–163)					
1^a^	F^d^	2 days	10 days	2 years	First cousins	Lethargy progressed to coma and seizure during the neonatal period. Speech delay since the age of 2 years	1,000	263	Moderate excretion	15	45	30	c.552-555delTTTC; p. (Phe185SerfsTer8)
										1.10			
2^a^	M^d^	3 days	1 month	5 years	First cousins	Lethargy progressed to coma, liver dysfunction and coagulopathy during the neonatal period. Currently, a normal neurological examination	700	101 Repeat at the age 6 months	Not detected	30	400	350	
								239		2.79			
3^a^	M	4 days	17 days	Deceased	First cousins	Lethargy progressed to coma, coagulopathy, and liver dysfunction during the neonatal period	625	N/A^d^	N/A^d^	24	140	150	
						Died at age 1 month				2.10			
4^b^	M	2 days	1 month	18 months	Not cousins but from the same village	Lethargy progressed to coma and seizure during the neonatal period. Growth failure, developmental delay, central hypotonia, lower limbs spasticity, and microcephaly since the age of 4 months	230	255	Moderate excretion	14.3	60	42	
										1.10			
5^b^	M	18 months	18 months	21 months	Not cousins but from the same village	Lethargy progressed to coma and seizure, coagulopathy, and liver dysfunction at age 18 months. Aversion to protein and speech delay at age since the age of 1 year	175	270	Massive excretion	28.4	520	552	
										2.04			
6^c^	M	4 years	4 years	12 years	First cousins once removed	Hepatomegaly, liver dysfunction, and coagulopathy were discovered accidentally at age 4 years. Frequent falls at 10 years. No signs of pyramidal tract dysfunction	194	250	Moderate excretion	27	108	152	
										3.94			
7^c^	M	3 years	19 years	30 years	First cousins	Muscle weakness and seizure at age 3 years. Ataxia, spastic paraparesis, hyperreflexia at age 19 years	373	326	Not detected	14.3	37	40	
										1.40			
8^c^	M	15 years	15 years	25 years	First cousins	Abnormal behaviour, lethargy, ataxia at age 15 years. Spastic paraparesis and frequent falls since the age of 17 years	194	272	Not detected	14.7	38	57	
										1.28			
9	M	7 years	18 years	20 years	First cousins	Developmental delay, learning disability and spastic gait at age 7 years. Spastic paraparesis, frequent falls, and aversion to protein since the age of 11 years	74	266	Moderate excretion	14.4	34	48	
10	M	13 days	27 days	10 years	First cousins	Lethargy, seizure, and coma at age 13 days	100	230	Moderate excretion	10.2	32	17	c.446delG, p. (Ser149ThrfsTer45)
						Developmental delay, spastic paraparesis, hypotonia at 5 years				0.91			
11	M	29 years	29 years	29 years	Second cousins	Poor school performance and left the school at age 14 years. Lethargy and coma at age 29 years. Normal cognition and gait	95	361	Moderate excretion	16	21	32	
										1.2			
12	M	2 years	2 ½ years	4 ½ years	First cousins	Seizure and speech delay at age 2 years. Currently has normal speech and cognition	203	307	Moderate excretion	17	161	460	
										1.4			
13	M	19 months	20 months	2 years	First cousins	Hepatomegaly and coagulopathy at 19 months. Well at 2 years	130	171	Moderate excretion	57	2,433	2,112	
										5.1			

Four patients, (patients 10–13, Table 1), born to first-cousin parents who belong to three different extended families, had a recurrent homozygous frameshift variant in the *SLC25A15* gene; Chr13: 41379385del, NM_014252.4:c.446delG, (p.Ser149ThrfsTer45)[GRCh37]. This variant is rare; it was not detected in the healthy population dataset (gnomAD v2.1.1 and v3.1.1 July 2022). The affected region is highly conserved in mammals (Figures 1D,E). The c.446del resulted in frameshift and alteration of the following 45 amino acids, followed by premature termination of translation, and is predicted to result in nonsense-mediated mRNA decay of the mutant transcripts.

### Demographic distribution

Eight out of the nine patients who were homozygous for the novel frameshift variant p. (Phe185SerfsTer8) in the *SLC25A15* gene, originated from a single village in the southern part of Palestine. Patients one to three were born to first-cousin parents who belong to the same extended family. Patients six to eight are also born to consanguineous first-cousin parents who belong to another extended family residing in the same village. Patients four and five are born to non-consanguineous parents living in the same village. Patient nine was born to first-cousin parents from another nearby village in the south.

All of the patients with the p. (Ser149ThrfsTer45) variant are born to first-cousin parents. The parents in the three nuclear families were not related and originated from three different communities. The communities are in distant geographical locations, and we were not able to establish shared historical migration origins between them.

### Clinical phenotype

In the first group of patients, homozygous for the p. (Phe185SerfsTer8), patients one to four presented in the neonatal period with lethargy progressing to coma, seizures, and hyperammonemia. Liver dysfunction and coagulopathy were reported in patients 2 and 3. Although severe hyperammonemia was reported in patients 1–3, (1,000 μmol/L, 700 μmol/L, and 625 µmol/L respectively), Patient 4 (serum ammonia 230 μmol/L) had the most severe phenotype with severe microcephaly, growth failure, central hypotonia, and lower limbs spasticity since the age of 4 months. The diagnosis of HHH was challenging for patient two; The family had two previous siblings deceased in the early neonatal period with encephalopathy and no obvious diagnosis. Patient two was followed tightly after birth, and he rapidly progressed to coma on day 3 of life with severe hyperammonemia, while his plasma ornithine level was initially normal (see Table 1). Empirical parenteral therapy with sodium benzoate and arginine was initiated, which resulted in an improvement in his status. Repeated ornithine measurement at 6 months of age was high and strongly suggested the diagnosis of HHH. Patient five presented acutely in early childhood with lethargy progressing to coma, seizure, liver failure, and hyperammonemia (serum ammonia 175 μmol/L). He also had an aversion to a protein diet and a speech delay that was noticed by his parents at the age of 1 year. Patient six was identified accidentally at the age of 4 years to have hepatomegaly, liver dysfunction, and coagulopathy. His most recent evaluation at the age of 11 years did not reveal signs of pyramidal tract dysfunction, although he complained of frequent falls. Serum ammonia was 194 μmol/L. The clinical phenotype of patients 7 and 8, who are brothers, and patient nine are characterized by slowly progressive motor dysfunction starting in late childhood and progressing to ataxia, spastic paraparesis, and mental regression. Patient nine also has an aversion to protein. The three patients had progressive pyramidal tract dysfunction. Overall, pyramidal tract dysfunction was reported in four patients in this group (patients 4, 7, 8, and 9).

The second group of patients, who are homozygous for the p. (Ser149ThrfsTer45) frameshift variant, had variable clinical features and symptom severity. Patient 10 had a neonatal onset of lethargy progressing to coma and seizure, and hypotonia. Serum ammonia was mildly elevated at the initial presentation (100 μmol/L). Developmental delay and spastic paraparesis were reported at the age of 5 years. Patient 11 presented for the first time at the age of 29 years when he developed an acute episode of lethargy progressing to coma. Serum ammonia at presentation was only mildly elevated (95 μmol/L). Upon recovery from the acute episode, the evaluation revealed acceptable cognitive functioning apart from historic mild learning deficits, normal neurological examination without signs of pyramidal tract dysfunction, and normal liver function tests. Patient 12 presented solely with seizures and a speech delay at the age of 2 years. Serum ammonia was 203 μmol/L. Currently, he is 4.5 years old and has fluent speech and normal cognition. Seizures stopped in the last 2 years. Patient 13 presented with hepatomegaly, liver dysfunction, and coagulopathy at the age of 19 months. Evaluation at the age of 2 years revealed normal cognition and neurological examination.

Plasma ornithine was elevated in all tested patients (except for patient three who deteriorated rapidly and died before the completion of the metabolic workup). Plasma ammonia was elevated in all patients except patient nine at the initial presentation. A qualitative assessment of urine homocitrulline was performed on six patients and was elevated in all of them. Qualitative analysis of urine orotic acid was performed in twelve patients, nine of them had an increased excretion.

Therapy with a low-protein diet, sodium benzoate, and l-citrulline was administered to all patients except patient three who died at 1 month. Patient 11 stopped therapy after 3 months of the diagnosis and was unavailable for follow-up. During follow-up, serum ammonia and liver transaminases remained normal in all patients.

## Discussion

HHH syndrome is a rare autosomal recessive disorder of the urea cycle characterized by impaired ornithine transport across the inner mitochondrial membrane (Camacho et al., 1999; Salvi et al., 2001). It is caused by disease-causing variants in the *SLC25A15* gene. Several pathogenic variants are responsible for the clinical phenotype. Reported variants include missense, nonsense, small insertion and deletions, and one gross deletion. HHH is characterized by high clinical variability ranging from the acute onset of seizures, coma, and hepatic signs to a mild form with learning difficulties and mild neurological involvement (Martinelli et al., 2015). Most patients with HHH syndrome develop signs of pyramidal tract dysfunction ranging from hyperreflexia, and spastic gait to spastic paraparesis (Salvi et al., 2001; Debray et al., 2008; Kim et al., 2012; Martinelli et al., 2015; Panza et al., 2019; Olivieri et al., 2019; Ebrahimi-Fakhari et al., 2021).

In this study, we retrospectively report on thirteen HHH syndrome patients recruited from the two major tertiary care metabolic units in Palestine. The diagnosis was established by the metabolic features of hyperammonemia, hyperornithinemia, and urinary excretion of homocitrulline and/or orotic acid. Targeted gene sequencing confirmed the diagnosis and identified two prevalent pathogenic variants in the *SLC25A15* gene inherited in a homozygous, autosomal recessive pattern. The p. (Phe185SerfsTer8) variant is a novel variant that was identified in nine out of thirteen patients (70% of patients). Eight out of the nine patients with this pathogenic variant belong to three different extended families, residing in one village. Seven out of the nine patients are products of consanguineous first-cousin union. The parents of the two remaining patients are not relatives, but they originate from the same village where other cases are reported.

In four out of thirteen patients (30% of patients), we identified a recurrent homozygous frameshift variant in the *SLC25A15* gene; p. (Ser149ThrfsTer45). The reported patients belong to three different nuclear families originating from three different and distant communities. In addition to our patient cohort, the c.446delG was previously reported in two Palestinian brothers presenting with variable degrees of delayed development and seizures (Korman et al., 2004), and in a third Palestinian patient who presented with spastic paraparesis, cerebellar ataxia, mild polyneuropathy in adulthood (Hengel et al., 2020).

The initial presentation, clinical phenotype, and time of diagnosis in HHH syndrome are highly variable, and this might cause diagnosis difficulties in some instances. The main clinical presentation of our patients was acute encephalopathy (in seven out of thirteen patients) requiring intensive supportive care and mechanical ventilation. One of the patients had his initial presentation with acute encephalopathy at the age of 29 years. This later acute presentation is not common in HHH but has already been reported (Tezcan et al., 2012; Filosto et al., 2013; Silfverberg et al., 2018). Three patients presented with acute encephalopathy and liver involvement including prolonged coagulation profile and elevated liver transaminases (patients 2, 3, and 5, Table 1). We noticed that acute encephalopathy and liver dysfunction improved with intensive supportive care, dietary management, and supplementation with sodium benzoate and l-citrulline. Thus, it is important to consider rare but treatable metabolic diseases, such as HHH syndrome, in acute encephalopathy in adults as well as in children.

Two patients had liver failure as the only primary presentation (patients 6 and 13, Table 1). In these two patients, treatment with sodium benzoate and dietary protein restriction improved liver functioning. This might suggest that intensive metabolic support is important in patients presenting acutely with encephalopathy or multiple system involvement. Chronic neurological manifestations were commonly detected among the patients (nine out of thirteen patients), with symptoms including speech and development delay, cognitive impairment, learning difficulties, seizures, pyramidal signs, and spastic paraparesis. Noticeably, spastic paraparesis developed in some patients during childhood (patients 9 and 10, Table 1), and was common in older patients beyond the age of 15 years (three out of four patients), irrespective of dietary management and treatment with sodium benzoate. Additionally, there was no correlation between the development of pyramidal tract dysfunction and the degree of hyperammonemia. These results support previous observations regarding the difficulty in preventing neurologic complications that remain a therapeutic challenge for HHH patients (Martinelli et al., 2015).

Targeted gene sequencing for the *SLC25A15* gene identified the underlying molecular cause of a complex multisystem disorder presenting with liver damage, encephalopathy and developmental delay. The combination of hyperornithinemia, hyperammonemia, and homocitrullinuria and increased levels of orotic acid in plasma or urine are specific for HHH. Hepatic encephalopathy improved with specific dietary management and supplementation with sodium benzoate, and l-citrulline. The molecular findings are consistent with the clinical and biochemical presentation. However, the possibility of having another coexistent genetic disease, which might be contributing to the heterogeneity of the clinical presentation can not be absolutely excluded. Next generation sequencing methods including whole exome sequencing and whole genome sequencing are the methods of choice in detecting pathogenic variants in other genes when suspected.

Patients presented from different villages and communities distributed throughout the West Bank, Palestine, and were recruited from the two metabolic centers; which cover a population of 3.19 millions according to the 2021 Palestinian Bureau of Stastictics records^1^. In this population, consanguineous marriages are common and account for about 40% of total marriages (Abu-Libdeh, B. and Teebi, A.S., 2010). Subsequently, autosomal recessive diseases, including metabolic diseases, are common in this population. The two metabolic centers receive referrals from several primary and secondary health-care services in Palestine, and provide access to clinical, biochemical, and molecular testing for a wide range of metabolic conditions. The clinical heterogeneity and the demographic distribution of the detected variants require special attention from the perspectives of the health care system. The two reported variants in the *SLC25A15* gene in the cohort were present in more than one extended family, and two or more villages and communities. Additionally, in two cases, patients were born to non-consanguineous parents originating from the same village where the variant was detected. This could suggest that these genetic variants might have a wider carrier distribution in this population and an older ancestral origin, and thus implicates the need of establishing a population screening program for the carrier status of the variants in the affected communities and villages as a first health care priority. Additionally, national neonatal screening programs are restricted to congenital hypothyroidism and phenylketonuria. The inclusion of other treatable or controllable metabolic and genetic diseases such as HHH in neonatal screening programs may help better identify newborns presenting with acute decompensation and encephalopathy early on, allowing for an improved diagnosis and management guidance, and the adoption of effective genetic counseling strategies for families of affected children.

Limitations to this study include the retrospective nature of the study design and the small sample size, reflecting the ultra-rare nature of HHH syndrome.

## Data Availability

The datasets for this article are not publicly available due to concerns regarding participant/patient anonymity. Requests to access the datasets should be directed to the corresponding author.

## Footnotes

1
https://www.pcbs.gov.ps/Downloads/book2595.pdf.

## Abbreviations

AST, Aspartate Transaminase; ALT, Alanine Transaminase; F, Female; INR, International Normalised Ratio; IU, International Unit; M, Male; N/A, Not Available; PT, Prothrombin Time

## References

[B1] Abu-Libdeh B. Teebi A. S. (2010). “Genetic disorders among the Palestinians,” in Genetic disorders among Arab populations. Editor Teebi A. (Berlin, Heidelberg: Springer).

[B2] Camacho J. A. Obie C. Biery B. Goodman B. K. Hu C. A. Almashanu S. (1999). Hyperornithinaemia-hyperammonaemia-homocitrullinuria syndrome is caused by mutations in a gene encoding a mitochondrial ornithine transporter. Nat. Genet. 22 (2), 151–158. 10.1038/9658 10369256

[B3] Debray F. G. Lambert M. Lemieux B. Soucy J. F. Drouin R. Fenyves D. (2008). Phenotypic variability among patients with hyperornithinaemia-hyperammonaemia-homocitrullinuria syndrome homozygous for the delF188 mutation in SLC25A15. J. Med. Genet. 45 (11), 759–764. 10.1136/jmg.2008.059097 18978333

[B4] Ebrahimi-Fakhari D. Saffari A. Pearl P. L. (2021). Childhood-onset hereditary spastic paraplegia and its treatable mimics. Mol. Genet. Metab. S1096-7192 (21), 00735–00736. 10.1016/j.ymgme.2021.06.006 PMC8843241 34183250

[B5] Filosto M. Alberici A. Tessa A. Padovani A. Santorelli F. M. (2013). Hyperornithinemia-hyperammonemia-homocitrullinuria (HHH) syndrome in adulthood: A rare recognizable condition. Neurol. Sci. 34 (9), 1699–1701. 10.1007/s10072-012-1266-8 23247599

[B6] Hengel H. Buchert R. Sturm M. Haack T. B. Schelling Y. Mahajnah M. (2020). First-line exome sequencing in Palestinian and Israeli Arabs with neurological disorders is efficient and facilitates disease gene discovery. Eur. J. Hum. Genet. 28 (8), 1034–1043. 10.1038/s41431-020-0609-9 32214227 PMC7382450

[B7] Hommes F. A. Roesel R. A. Metoki K. Hartlage P. L. Dyken P. R. (1986). Studies on a case of HHH-syndrome (hyperammonemia, hyperornithinemia, homocitrullinuria). Neuropediatrics 17 (1), 48–52. 10.1055/s-2008-1052499 3960284

[B8] Inoue I. Saheki T. Kayanuma K. Uono M. Nakajima M. Takeshita K. (1988). Biochemical analysis of decreased ornithine transport activity in the liver mitochondria from patients with hyperornithinemia, hyperammonemia and homocitrullinuria. Biochim. Biophys. Acta 964 (1), 90–95. 10.1016/0304-4165(88)90071-2 3334877

[B9] Kim S. Z. Song W. J. Nyhan W. L. Ficicioglu C. Mandell R. Shih V. E. (2012). Long-term follow-up of four patients affected by HHH syndrome. Clin. Chim. Acta. 413 (13-14), 1151–1155. 10.1016/j.cca.2012.03.015 22465082

[B10] Korman S. H. Kanazawa N. Abu-Libdeh B. Gutman A. Tsujino S. (2004). Hyperornithinemia, hyperammonemia, and homocitrullinuria syndrome with evidence of mitochondrial dysfunction due to a novel SLC25A15 (ORNT1) gene mutation in a Palestinian family. J. Neurol. Sci. 218 (1-2), 53–58. 10.1016/j.jns.2003.10.017 14759633

[B11] Martinelli D. Diodato D. Ponzi E. Monné M. Boenzi S. Bertini E. (2015). The hyperornithinemia-hyperammonemia-homocitrullinuria syndrome. Orphanet J. Rare Dis. 10, 29. 10.1186/s13023-015-0242-9 25874378 PMC4358699

[B12] Olivieri G. Pro S. Diodato D. Di Capua M. Longo D. Martinelli D. (2019). Corticospinal tract damage in HHH syndrome: A metabolic cause of hereditary spastic paraplegia. Orphanet J. Rare Dis. 14 (1), 208. 10.1186/s13023-019-1181-7 31443672 PMC6708179

[B13] Oyanagi K. Tsuchiyama A. Itakura Y. Sogawa H. Wagatsuma K. Nakao T. (1983). The mechanism of hyperammonaemia and hyperornithinaemia in the syndrome of hyperornithinaemia, hyperammonaemia with homocitrullinuria. J. Inherit. Metab. Dis. 6 (3), 133–134. 10.1007/BF01800748 6422148

[B14] Panza E. Martinelli D. Magini P. Dionisi Vici C. Seri M. (2019). Hereditary spastic paraplegia is a common phenotypic finding in ARG1 deficiency, P5CS deficiency and HHH syndrome: Three inborn errors of metabolism caused by alteration of an interconnected pathway of glutamate and urea cycle metabolism. Front. Neurol. 10, 131. 10.3389/fneur.2019.00131 30853934 PMC6395431

[B15] Salvi S. Santorelli F. M. Bertini E. Boldrini R. Meli C. Donati A. (2001). Clinical and molecular findings in hyperornithinemia-hyperammonemia-homocitrullinuria syndrome. Neurology 57 (5), 911–914. 10.1212/wnl.57.5.911 11552031

[B16] Schwarz J. M. Rödelsperger C. Schuelke M. Seelow D. (2010). MutationTaster evaluates disease-causing potential of sequence alterations. Nat. Methods 7 (8), 575–576. 10.1038/nmeth0810-575 20676075

[B17] Shimizu H. Maekawa K. Eto Y. (1990). Abnormal urinary excretion of polyamines in HHH syndrome (hyperornithinemia associated with hyperammonemia and homocitrullinuria). Brain Dev. 12 (5), 533–535. 10.1016/s0387-7604(12)80222-1 2288388

[B18] Silfverberg T. Sahlander F. Enlund M. Oscarson M. Hårdstedt M. (2018). Late onset hyperornithinemia-hyperammonemia-homocitrullinuria syndrome - how web searching by the family solved unexplained unconsciousness: A case report. J. Med. Case Rep. 12 (1), 274. 10.1186/s13256-018-1794-9 30243302 PMC6151189

[B19] Smith L. Lambert M. A. Brochu P. Jasmin G. Qureshi I. A. Seidman E. G. (1992). Hyperornithinemia, hyperammonemia, homocitrullinuria (HHH) syndrome: Presentation as acute liver disease with coagulopathy. J. Pediatr. Gastroenterol. Nutr. 15 (4), 431–436. 10.1097/00005176-199211000-00011 1469525

[B20] Tezcan K. Louie K. T. Qu Y. Velasquez J. Zaldivar F. Rioseco-Camacho N. (2012). Adult-onset presentation of a hyperornithinemia-hyperammonemia-homocitrullinuria patient without prior history of neurological complications. JIMD Rep. 3, 97–102. 10.1007/8904_2011_71 23430880 PMC3509867

[B21] Tsujino S. Kanazawa N. Ohashi T. Eto Y. Saito T. Kira J. (2000). Three novel mutations (G27E, insAAC, R179X) in the ORNT1 gene of Japanese patients with hyperornithinemia, hyperammonemia, and homocitrullinuria syndrome. Ann. Neurol. 47 (5), 625–631. 10.1002/1531-8249(200005)47:5<625::aid-ana10>3.0.co;2-q 10805333

[B22] Wild K. T. Ganetzky R. D. Yudkoff M. Ierardi-Curto L. (2019). Hyperornithinemia, hyperammonemia, and homocitrullinuria syndrome causing severe neonatal hyperammonemia. JIMD Rep. 44, 103–107. 10.1007/8904_2018_132 30187369 PMC6323011

[B23] Zhou H. Gao M. Skolnick J. (2018). ENTPRISE-X: Predicting disease-associated frameshift and nonsense mutations. PloS one 13 (5), e0196849. 10.1371/journal.pone.0196849 29723276 PMC5933770

